# Structure and Functional Characteristics of Soybean Protein from Different Northeast Cultivars and Their Effects on the Quality of Soymilk Gel

**DOI:** 10.3390/foods14234029

**Published:** 2025-11-24

**Authors:** Xiaoyu Xia, Chunlei Zhang, Shiyao Zhang, Tianjiao Gao, Shuping Yan, Xiuqing Zhu, Jiaxin Kang, Guixing Zhao, Sobhi F. Lamlom, Honglei Ren, Jiajun Wang

**Affiliations:** 1 Soybean Research Institute, Heilongjiang Academy of Agricultural Sciences, Harbin 150086, Chinasobhifaid@alexu.edu.eg (S.F.L.); 2 College of Food Engineering, Harbin University of Commerce, Harbin 150028, China; 3 College of Food and Biological Engineering, Qiqihar University, Qiqihar 161006, China; 4 Plant Production Department, Faculty of Agriculture Saba Basha, Alexandria University, Alexandria 21531, Egypt

**Keywords:** soybean varieties, soymilk gel, tofu, protein composition, 11S/7S ratio, protein structure, gel properties, texture, microstructure, variety selection

## Abstract

Soymilk gel quality hinges on soybean protein composition and structure, yet direct comparisons linking protein traits to gel properties are limited. This study compared seven Northeast Chinese soybean varieties to identify which protein characteristics best predict tofu gel quality. Protein analysis included composition (11S/7S ratio), structure, and functional properties. Gel quality was measured through yield, water retention, texture, rheology, and microstructure imaging. Results showed substantial variation among varieties: 11S/7S ratios ranged from 1.14 to 4.10, solubility from 57.50% to 69.74%, gel yield from 193.25% to 236.12%, water-holding capacity from 42.09% to 60.23%, and gel firmness from 1520 to 1889 gf. The 11S/7S ratio emerged as the strongest quality predictor, correlating with gel firmness (R = 0.92) and elasticity (R = 0.98), while solubility correlated with yield (R = 0.79) and water retention (R = 0.83). Microscopy revealed that variety HD-1, with the highest 11S/7S ratio (4.10) and solubility (69.74%), formed dense networks with small pores (20–50 μm), whereas variety HK-60 (ratio 1.14) produced coarse structures with large pores (100–200 μm). HD-1 showed the best overall performance. Varieties with 11S/7S ratios above 3.5 and solubility above 68% consistently produced high-quality gels, while ratios below 2.5 indicated poor gel formation regardless of total protein content. These findings demonstrate that protein composition matters more than protein quantity for tofu quality. The approach enables rapid variety screening and provides practical guidelines for tofu manufacturers and soybean breeders.

## 1. Introduction

Soybean (*Glycine max* L.) has been cultivated in China for over 5000 years and remains a critical source of plant-based protein globally [1]. Soymilk gel, or tofu, is produced through thermal denaturation and salt-induced coagulation of soy proteins. It is the most economically significant traditional soy product, with worldwide consumption exceeding 15 million metric tons annually [2,3]. The quality of soymilk gel depends fundamentally on the gelation behavior of soy storage proteins, which form three-dimensional networks through disulfide bonding, hydrophobic interactions, and electrostatic forces [4,5].

Soy storage proteins consist primarily of 11S globulin (glycinin) and 7S β-conglycinin, collectively comprising 70–80% of total seed protein [6]. The 11S fraction, a hexamer with abundant cysteine residues, provides gel rigidity and hardness through extensive disulfide cross-linking [7]. In contrast, the 7S fraction, a glycosylated trimer with lower cysteine content, contributes flexibility and water-binding capacity [8]. Multiple studies have established that the 11S/7S ratio critically influences gel properties: higher ratios correlate with increased hardness, yield, and water holding capacity (WHC), while lower ratios produce softer, more elastic gels [8,9]. Beyond composition, protein secondary structure has a significant impact on functionality. Zheng et al. [10] demonstrated that β-sheet content positively impacts gel hardness through enhanced intermolecular hydrogen bonding, while high α-helix content limits protein unfolding during thermal processing. Fluorescence spectroscopy studies reveal that tertiary structure stability, reflected in tryptophan residue burial within hydrophobic cores, influences protein behavior during heat-induced gelation [11]. Functional properties, particularly solubility and gelation capacity, serve as intermediate indicators linking protein structure to final gel quality, with high solubility enabling the formation of uniform, fine-pored gel networks [12].

Soybean varieties show significant genetic diversity in protein content (35–45%), 11S/7S ratios (1.0–4.5), and structural traits [13]. This variation reflects adaptation to different environments and breeding goals, mainly focused on agronomic traits and total protein content rather than processing qualities. Northeast China, especially Heilongjiang Province, produces over 40% of China’s soybeans, with varieties suited for short growing seasons and valued for high protein [14,15]. The region was selected for several strategic reasons: First, regional importance, as Northeast China produces more than 40% of the country’s soybeans, with Heilongjiang contributing about 30% [14], making it China’s largest soybean-producing area and a major supplier for tofu manufacturing nationwide. Second, unique germplasm features: Northeast varieties, adapted to the temperate zone with a short growing season of 110–130 frost-free days, have been bred for high protein levels (around 38–42%) and cold tolerance, resulting in distinct protein profiles [16]. Third, industrial relevance: Traditional Chinese tofu producers favor Northeast soybeans due to their reputed superior gel-forming properties, although detailed validation of processing traits is limited [17]. Fourth, genetic variation within adaptation: Even under similar environmental conditions, Northeast soybean varieties show considerable variation in 11S/7S ratios (initial tests suggest a range from 1.0 to 4.5), making them excellent for structure-function studies [18]. Lastly, practical significance: Research on Northeast varieties can directly benefit China’s most significant segment of the tofu industry, and the analytical methods developed here can be applied to other regional germplasm collections globally [19]. By focusing on this economically and regionally important germplasm, our study addresses both scientific questions and practical challenges in tofu production.

Despite extensive research into soy protein gelation, significant knowledge gaps hinder its practical use in variety selection and breeding. Most studies focus on isolated 11S and 7S components or on single varieties under different processing conditions, complicating comparisons of results and recommendations for tofu producers [20,21]. Although the importance of the 11S/7S ratio is acknowledged, comprehensive datasets comparing various commercial varieties under the same processing conditions are limited, especially for Northeast Chinese cultivars, which dominate tofu production [19]. Integrated structural analyses—combining protein composition (SDS-PAGE), secondary structure (FTIR), tertiary structure (fluorescence spectroscopy), and functional properties—are rare. Most research examines only one or two aspects, making it challenging to predict gel quality based on protein traits [8,22]. SEM has been used to observe the microstructure of tofu gels, but systematic comparisons linking specific protein traits to network architecture and functionality across varieties are scarce [23]. Although some correlations between protein traits and gel properties exist, comprehensive datasets across multiple varieties for predictive modeling are missing [24,25]. This gap in understanding leads tofu producers to rely on costly, time-consuming trial-and-error approaches, resulting in inconsistent quality and wasted resources [25].

This study fills existing gaps by thoroughly analyzing seven representative Northeast Chinese soybean varieties. It investigates protein composition (11S/7S ratios by SDS-PAGE), secondary structure (FTIR), tertiary structure (fluorescence spectroscopy), and functional properties, such as solubility, water/oil holding capacity, and gelation ability, under consistent conditions. The quality of soymilk gel is evaluated from multiple perspectives, including yield, WHC, texture profile analysis, dynamic rheology, and microstructure (SEM). Correlation analysis identifies quantitative relationships between protein traits and product characteristics. Results reveal that the 11S/7S ratio is the most dependable predictor of gel quality, strongly correlating with gel hardness (R = 0.92) and storage modulus (R = 0.98). Protein solubility also shows a strong correlation with gel yield (R = 0.79) and WHC (R = 0.83). SEM images demonstrate that varieties with high 11S/7S ratios and solubility (HD-1: 4.10, 69.74%) form dense, uniform networks with small pores (20–50 μm). In contrast, varieties with low ratios (HK-60: 1.14) develop coarse structures with large voids (100–200 μm), explaining their different functional properties. HD-1 emerges as the best variety, with the highest 11S/7S ratio (4.10), solubility (69.74%), gel yield (236.12%), WHC (60.23%), and improved texture. Based on these results, we suggest selecting varieties with 11S/7S ratios above 3.5 and solubility above 68% to produce high-quality gels. Ratios below 2.5 indicate weak gelation potential, regardless of total protein content. This comprehensive approach connects protein science with industry applications, providing mechanistic insights into gelation. It also offers valuable tools for rapid variety screening, quality assessment, and breeding programs focused on processing properties rather than just protein levels.

## 2. Materials and Methods

### 2.1. Soybean Samples

Seven soybean varieties (*Glycine max* L.) representative of Northeast China’s fourth temperate zone production were obtained from the Heilongjiang Academy of Agricultural Sciences germplasm collection (Harbin, China): HJ-2, HD-1, HK-59, HK-60, HH-35, HH-43, and HH-45. All varieties were harvested in 2023 from the same experimental field (45°41′ N, 126°38′ E, Harbin) under identical agronomic management to minimize environmental variation. Seeds were cleaned, dried to 12% moisture content, and stored at 4 °C in sealed containers until analysis. To control batch effects, all soymilk and gel preparations were performed using seeds from the same harvest batch for each variety, processed in parallel under identical laboratory conditions. Laboratory temperature and relative humidity were maintained at 22 ± 1 °C and 50 ± 5% RH, respectively, during all processing steps. Each variety was prepared in triplicate independent batches, and all experiments were repeated on different days to ensure reproducibility. Seed quality was verified to meet the Chinese National Standard for food-grade soybean [26].

### 2.2. Compositional Analysis of Soybeans

#### 2.2.1. Protein Content

Crude protein content was determined in triplicate by the Kjeldahl method following Chinese National Standard GB 5009.5-2016 [27]. Approximately 1.0 g of finely ground soybean powder (100 mesh) was digested with concentrated sulfuric acid (98%, *w*/*v*) using copper sulfate and potassium sulfate as catalysts. After neutralization and distillation, nitrogen content was quantified by titration with standardized HCl (0.1 mol/L). Protein content was calculated using a conversion factor of 6.25 (N × 6.25). Results are expressed as g/100 g dry weight basis.

#### 2.2.2. Fat Content

Crude fat was extracted and quantified using the Soxhlet extraction method according to GB 5009.6-2016 [28]. Ground soybean samples (5.0 g) were extracted with petroleum ether (boiling point 30–60 °C) for 8 h in a Soxhlet apparatus (Buchi Extraction System B-811, Flawil, Switzerland). After extraction, the solvent was evaporated under reduced pressure, and the residue was dried at 105 °C to constant weight. Fat content is reported as g/100 g dry weight basis.

#### 2.2.3. Isoflavone Content

Total isoflavone content (daidzin, genistin, daidzein, and genistein) was determined by high-performance liquid chromatography (HPLC) following GB/T 23788-2009 [29]. Samples (2.0 g) were extracted with 80% aqueous methanol (*v*/*v*) at room temperature for 2 h with continuous stirring. After centrifugation (8000× *g*, 10 min), supernatants were filtered through 0.45 μm PVDF membranes. Analysis was performed on an Agilent 1260 Infinity HPLC system (Agilent Technologies, Santa Clara, CA, USA) equipped with a diode array detector and a C18 column (4.6 × 250 mm, 5 μm, Agilent). Mobile phase consisted of acetonitrile (A) and 0.1% acetic acid in water (B) with gradient elution: 0–30 min, 15–40% A. Flow rate was 1.0 mL/min, injection volume 20 μL, column temperature 30 °C, and detection wavelength 260 nm. Results are expressed as μg/g dry weight using external standard calibration.

#### 2.2.4. Selenium Content

Selenium was quantified by atomic fluorescence spectrometry (AFS) following GB 5009.93-2017 [30]. Samples (0.5 g) were digested with a nitric acid (65%, *v*/*v*) and perchloric acid (70%, *v*/*v*) mixture (4:1, *v*/*v*) at 180 °C until clear. After appropriate dilution, selenium content was measured using an AFS-9230 atomic fluorescence spectrometer (Beijing Jitian Instrument Co., Ltd., Beijing, China) with hydride generation. Operating conditions: lamp current 60 mA, atomizer temperature 800 °C, carrier gas (argon) flow rate 400 mL/min. Results are reported as μg/100 g dry weight.

#### 2.2.5. Phytic Acid Content

Phytic acid was determined by ion-exchange chromatography according to GB 5009.153-2016 [31]. Samples (2.0 g) were extracted with 0.5 mol/L HCl (25 mL) for 16 h at room temperature with continuous shaking. Extracts were filtered and analyzed on a Dionex ICS-5000 ion chromatography system (Thermo Fisher Scientific, Waltham, MA, USA) equipped with a CarboPac PA200 column (3 × 250 mm). Gradient elution employed deionized water (A) and 150 mmol/L NaOH (B): 0–5 min, 100% A; 5–25 min, 0–100% B; 25–35 min, 100% B. Flow rate was 0.3 mL/min and detection used suppressed conductivity. Results are expressed as mg/g dry weight.

### 2.3. Protein Extraction

Soy protein isolate (SPI) was prepared by alkaline extraction followed by isoelectric precipitation as described by Hu et al. [32] with modifications. Whole soybeans were ground to pass through a 100-mesh sieve (150 μm) using a laboratory mill (FW100, Taisite Instrument Co., Ltd., Tianjin, China). Lipids were removed by shaking defatted soybean flour with *n*-hexane (1:3, *w*/*v*) for 10 h at room temperature, followed by air-drying in a fume hood for 48 h to remove residual solvent. Defatted flour (50 g) was dispersed in Tris-HCl buffer (750 mL, 0.05 mol/L, pH 8.2) and stirred at 45 °C for 1 h using a temperature-controlled stirrer (500 rpm). The dispersion was centrifuged at 4000× *g* for 30 min at 20 °C (Allegra X-30R, Beckman Coulter, Brea, CA, USA). The supernatant was collected and pH adjusted to 4.5 using 1 mol/L HCl to precipitate proteins at their isoelectric point. The mixture was stored overnight at 4 °C to ensure complete precipitation. After thorough mixing, the precipitate was collected by centrifugation (4000× *g*, 30 min, 20 °C) and washed three times with deionized water to remove salts and soluble carbohydrates. The washed precipitate was neutralized to pH 7.0 using 1 mol/L NaOH, frozen at −80 °C for 48 h, and freeze-dried (ALPHA 1-2 LD plus, Martin Christ, Osterode, Germany) for 48 h at −50 °C and 0.01 mbar. Dried protein powder was ground to pass through 100-mesh sieve and stored at −20 °C in vacuum-sealed bags until analysis. Protein purity was verified to be >90% by the Bradford assay [33].

### 2.4. Protein Characterization

#### 2.4.1. SDS-Polyacrylamide Gel Electrophoresis (SDS-PAGE)

Protein subunit composition was analyzed by SDS-PAGE following the method of Hu et al. [32] with minor modifications. Freeze-dried protein (10 mg) was dissolved in 10 mL PBS (0.02 mol/L, pH 7.0) to prepare 1 mg/mL stock solutions.

Non-reducing conditions: Sample solution (0.5 mL) was mixed with sample buffer (0.5 mL, containing 0.2 mg/mL SDS) and boiled for 5 min. After cooling, 20 μL was loaded onto gels.

Reducing conditions: Sample solution (0.5 mL) was mixed with sample buffer (0.5 mL) containing 0.2 mg/mL SDS and 50 μL β-mercaptoethanol (0.01 mol/L), boiled for 5 min, cooled, and 20 μL was loaded.

Electrophoresis was performed using a Mini-PROTEAN Tetra Cell system (Bio-Rad, Hercules, CA, USA) with 12% (*w*/*v*) separating gel and 5% (*w*/*v*) stacking gel. Running conditions: 80 V through stacking gel, 120 V through separating gel until bromophenol blue tracking dye reached the gel bottom (~90 min). A pre-stained protein ladder (10–245 kDa, Thermo Fisher Scientific, Waltham, MA, USA) served as molecular weight marker. Gels were stained with Coomassie Brilliant Blue G-250 solution (25% methanol, 10% acetic acid, 0.03% G-250, *v*/*v*/*w*) for 2 h with gentle shaking, then destained with methanol/acetic acid/water (20:10:70, *v*/*v*/*v*) until bands were clearly visible. Gels were imaged using a ChemiDoc XRS+ gel documentation system (Bio-Rad). Band intensity was quantified using ImageJ software (version 1.53k, National Institutes of Health, Bethesda, MD, USA) [34]. The 11S/7S ratio was calculated from the sum of band intensities for 11S subunits (A and B chains) divided by the sum for 7S subunits (α, α′, and β chains).

#### 2.4.2. Fourier Transform Infrared (FTIR) Spectroscopy

Secondary structure was analyzed by FTIR spectroscopy according to Xiang et al. [35]. Freeze-dried protein powder (2 mg) was placed on the diamond crystal of an attenuated total reflectance (ATR) accessory in a Spectrum Two FTIR spectrometer (PerkinElmer, Waltham, MA, USA). The pressure arm was rotated to compress the sample with a force gauge pressure set at 56 units. Spectra were collected from 4000 to 400 cm^−1^ at 4 cm^−1^ resolution with 32 scans per spectrum. Background air spectra were collected before each sample measurement. Raw spectra were processed using Spectrum software (version 10.6.2, PerkinElmer): ATR correction, baseline correction (rubber band method with 64 points), smoothing (Savitzky–Golay, 9 points), and normalization (min-max method). The amide I region (1700–1600 cm^−1^) was extracted and subjected to second-derivative analysis followed by Fourier self-deconvolution using PeakFit software (version 4.12, SeaSolve Software, Framingham, MA, USA). Gaussian curve fitting was performed to deconvolute overlapping bands. Secondary structure components were assigned as follows: α-helix (1650–1660 cm^−1^), β-sheet (1618–1640 cm^−1^ and 1670–1690 cm^−1^), β-turn (1660–1700 cm^−1^), and random coil (~1645 cm^−1^). Relative content of each structure was calculated as the percentage of individual peak area to total amide I band area.

#### 2.4.3. Fluorescence Spectroscopy

Intrinsic fluorescence was measured to assess the tertiary structure, following the method described by Chen et al. [36]. Protein powder (0.5 mg) was dissolved in 1.0 mL phosphate buffer (0.05 mol/L, pH 7.0) at 25 °C and centrifuged (8000× *g*, 5 min) to remove insoluble particles. Fluorescence emission spectra were recorded using a Lambda 365 UV-Vis spectrophotometer (PerkinElmer, Waltham, MA, USA) equipped with a fluorescence accessory. The excitation wavelength was 280 nm (5 nm bandwidth), and the emission was scanned from 300 to 450 nm (5 nm bandwidth) at a scan rate of 600 nm/min. Spectra were collected at 25 °C using 10 mm quartz cuvettes. Buffer blanks were subtracted from all spectra. The maximum emission wavelength (λ_max_) and fluorescence intensity at λ_max_ were recorded. Three replicate measurements were performed per sample.

#### 2.4.4. Protein Solubility

Solubility was determined using the method described by Mathias et al. [37] with modifications. Protein powder (100 mg) was dispersed in 100 mL PBS (0.01 mol/L, pH 7.0) and stirred at 90 rpm for 2 h at 25 °C. The dispersion was centrifuged (4000× *g*, 20 min, 20 °C), and the supernatant was carefully collected. Soluble protein concentration was determined by the Bradford method [33] at 595 nm using bovine serum albumin as a standard. Solubility was calculated as:

Solubility (%) = (Soluble protein/Total protein) × 100

Results are expressed as a percentage on a dry matter basis. All measurements were performed in triplicate.

#### 2.4.5. Water and Oil Retention Capacity

Water holding capacity (WHC) and oil holding capacity (OHC) were measured according to the method described by Sánchez et al. [38] with modifications. Protein powder (0.2 g) was accurately weighed into pre-weighed 15 mL centrifuge tubes. For WHC, 4 mL deionized water was added; for OHC, 4 mL soybean oil (Luhua Group Co., Ltd., Laiyang, Shandong, China) was added. Tubes were vortexed for 30 s, allowed to stand for 1 h at room temperature, then centrifuged (4000× *g*, 20 min, 20 °C). Supernatants were carefully decanted, and tubes were inverted on filter paper for 30 min to drain excess liquid. Tubes with hydrated/oiled protein were weighed.

WHC or OHC (g/g) = (*m*_1_ − *m*_2_)/*m*

where *m*_1_ is the mass of the tube with hydrated/oiled protein (g), *m*_2_ is the mass of the empty tube (g), and *m* is the mass of dry protein (g). Results represent grams of water or oil absorbed per gram of protein. Measurements were performed in triplicate.

#### 2.4.6. Gelation Capacity Assessment

The relative gelation capacity was estimated from 7S β-subunit and 11S B-polypeptide content in SDS-PAGE analysis under reducing conditions, as these subunits form the hydrophobic core during gel network formation [21,39]. Band densities were quantified using ImageJ software. Higher combined β-subunit and B-polypeptide intensity indicates greater gelation potential.

### 2.5. Soymilk Gel Preparation

Soymilk gels were prepared following the traditional Chinese tofu-making process described by Poysa et al. [40] with modifications to ensure reproducibility. Clean, whole soybeans (1.0 kg) were soaked in deionized water (3 L) at 25 ± 2 °C for 12 h. After soaking, beans were rinsed three times and drained thoroughly. Soaked beans and deionized water (6 kg, based on original dry bean weight) were ground together for 2 min using a commercial soymilk grinder (FSM-150, Xingaoke Food Machinery, Shenyang, China) equipped with a 100-mesh filter screen. Raw soymilk and okara (residue) were separated automatically through different outlets. The soymilk was further filtered through 100-mesh cloth to remove fine particles. The protein content of the filtered soymilk was determined to be 4.56 ± 0.12% (*w*/*v*) by the Kjeldahl method, where the variation (±0.12%) represents differences among the seven soybean varieties rather than technical replication error. Soymilk was heated to boiling (97 °C) in a stainless-steel vessel with continuous stirring to prevent scorching, then maintained at boiling for 15 min to ensure complete protein denaturation and eliminate beany flavor. The coagulant solution was prepared by dissolving MgCl_2_·6H_2_O (30 g) in deionized water (1 L) to obtain 3% (*w*/*v*) concentration. After boiling, soymilk was cooled with gentle stirring to 85 °C. The coagulant solution was added gradually while stirring continuously for 20 s to ensure uniform distribution. The mixture was allowed to stand undisturbed at room temperature (25 ± 2 °C) for 30 min to enable curd formation. The coagulated curd was carefully transferred to rectangular plastic molds (L × W × H: 15 × 15 × 9 cm) lined with cheesecloth. Pressing was performed using a custom-designed pressing apparatus applying 8 g/cm^2^ pressure (1.8 kg distributed over mold surface area) for 60 min to express whey while consolidating the protein network. Pressing continued until no additional whey was expelled. Formed tofu blocks were removed from molds and cut into uniform pieces (L × W × H: 4 × 5 × 2 cm) for analysis. Three independent batches were prepared for each variety.

### 2.6. Soymilk Gel Quality Evaluation

#### 2.6.1. Gel Yield

Gel yield was calculated according to Poysa et al. [40] as:

Yield (%) = (*M*_2_/*M*_1_) × 100

where *M*_1_ is the mass of fresh soybeans before soaking (g) and *M*_2_ is the mass of pressed gel after whey removal (g). Results represent the efficiency of protein conversion to gel product. Three batches per variety were analyzed.

#### 2.6.2. Water Holding Capacity

Gel WHC was determined following Chen et al. [41] with modifications. Gel samples (2.0 g, *W*_1_) were weighed accurately and centrifuged at 10,000× *g* for 20 min at 4 °C using a refrigerated centrifuge (GL21M, Xiangyi, Changsha, China). After centrifugation, samples were gently removed, surface moisture was blotted with filter paper, and samples were reweighed (*W*_2_).

WHC (%) = (*W*_2_/*W*_1_) × 100

Higher WHC indicates stronger water-binding capacity and gel stability. Six replicate measurements were performed per batch.

#### 2.6.3. Texture Profile Analysis (TPA)

Texture was evaluated using a TA.XT Plus texture analyzer (Stable Micro Systems, Godalming, UK) equipped with a 50 kg load cell and cylindrical probe (P/36R, 36 mm diameter) following the method of Liu et al. [42]. Gel samples were cut into cylinders (diameter 20 mm × height 20 mm) using a cork borer and allowed to equilibrate to room temperature (25 °C) for 30 min before testing. Test parameters: pre-test speed 5.0 mm/s, test speed 1.0 mm/s, post-test speed 5.0 mm/s, compression distance 40% of original height, trigger force 5 g, and 5 s interval between two compression cycles. Seven texture parameters were calculated from force-time curves using Exponent software (version 6.1.16, Stable Micro Systems): hardness (maximum force during first compression, gf), springiness (ratio of time during second compression to time during first compression), adhesiveness (negative area after first compression, gf·mm), chewiness (hardness × springiness × cohesiveness, gf), gumminess (hardness × cohesiveness, gf), cohesiveness (ratio of positive area during second compression to positive area during first compression), and resilience (ratio of upstroke energy during first compression to downstroke energy) [20]. Ten replicate measurements were performed per batch.

#### 2.6.4. Rheological Analysis

Dynamic oscillatory rheology was performed using a Discovery HR-2 rheometer (TA Instruments, New Castle, DE, USA) with parallel plate geometry (40 mm diameter, 1.0 mm gap) [43,44]. Gel samples were carefully cut into 1.0 mm thick disks using a precision slicer and placed on the lower plate, which was preheated to 25 °C. Excess sample was trimmed, and the edge was sealed with low-viscosity silicone oil to prevent dehydration during measurement. Strain sweep tests (0.01–100% strain, 1 Hz frequency) were first performed to identify the linear viscoelastic region (LVR). Subsequently, frequency sweep tests were conducted at 0.5% strain (within LVR) over 0.1–100 rad/s frequency range at 25 °C. Storage modulus (*G*′, representing elastic behavior) and loss modulus (*G*″, representing viscous behavior) were recorded as functions of frequency. Three replicate measurements were performed per batch.

#### 2.6.5. Scanning Electron Microscopy (SEM)

Gel microstructure was examined by SEM following Lee et al. [45] with modifications. Gel samples were cut into small cubes (2 × 5 × 2 mm) and fixed in 2.5% (*v*/*v*) glutaraldehyde solution (pH 6.8) at 4 °C for 1.5 h. Fixed samples were rinsed three times (10 min each) with 0.1 mol/L PBS (pH 6.8) to remove excess fixative. Dehydration was performed through a graded ethanol series: 50%, 70%, 90%, and 100% ethanol (*v*/*v*) for 15 min each. Samples were then transferred to ethanol/*tert*-butanol (1:1, *v*/*v*) for 20 min, followed by pure *tert*-butanol for 20 min. After dehydration, samples were frozen at −20 °C for 30 min and freeze-dried (ALPHA 1-2 LD plus, Martin Christ, Osterode, Germany) for 4 h at −50 °C and 0.01 mbar. Dried samples were mounted on aluminum stubs with conductive carbon tape and sputter-coated with gold (15 nm thickness) using an ion sputtering device (MSP-1S, Vacuum Device, Ibaraki, Japan). Coated samples were examined using a scanning electron microscope (S-3400N, Hitachi, Tokyo, Japan) operated at 15 kV accelerating voltage. Images were captured at 3000× magnification. Three samples per variety were examined, with multiple fields viewed per sample.

### 2.7. Statistical Analysis

All experiments were performed in triplicate unless otherwise stated, and data are presented as mean ± standard deviation (SD). Statistical analysis was conducted using SPSS software (version 22.0, IBM, Chicago, IL, USA). One-way analysis of variance (ANOVA) was performed to test for significant differences among varieties, followed by Duncan’s multiple range test for post hoc pairwise comparisons. Significance level was set at *p* < 0.05. Pearson correlation analysis was performed to evaluate linear relationships between protein characteristics and gel quality parameters. Correlation coefficients (*R*) and significance levels (*p*) are reported. Graphs were prepared using Origin software (version 2018, OriginLab, Northampton, MA, USA). Coefficients of variation (CV%) were calculated as (SD/mean) × 100 to assess variability across varieties. For compositional and functional property data, individual variety values represent means ± SD of three technical replicates (n = 3), reflecting measurement precision. Summary statistics (average values) reported across all seven varieties are calculated as the arithmetic mean of the seven variety means, with standard deviations computed from these variety means (n = 7) to represent between-variety biological variation rather than within-variety technical measurement error.

## 3. Results

### 3.1. Protein, Fat, and Bioactive Compound Profiles Across Soybean Germplasm

The compositional profiles of the seven Northeast Chinese soybean varieties revealed substantial genetic diversity across all measured parameters (Table 1). Protein content distinguished the varieties into four statistical groups. HK-60 contained significantly more protein (41.39 g/100 g) than all other varieties, representing a 16% increase over the lowest varieties, HJ-2 and HH-35 (35.56–35.69 g/100 g). All seven varieties qualified as high-protein soybeans (>35% dry weight basis). The intermediate protein varieties formed overlapping statistical groups: HH-45 and HK-59 (37.61–37.62 g/100 g, group b) did not differ from each other, while HD-1 and HH-43 (36.63–36.77 g/100 g, group c) also formed a statistically similar pair. Fat content varied 39% across varieties, from 16.04 g/100 g in HJ-2 to 22.36 g/100 g in HH-43. HH-43 and HD-1 formed the high-fat group (21.43–22.36 g/100 g), containing significantly more fat than HJ-2, HH-35, and HK-60. HK-59 exhibited intermediate fat levels (20.92 g/100 g) that did not differ significantly from those of either the high-fat varieties (HD-1, HH-43) or the mid-range varieties (HK-60, HH-45). Isoflavone content showed the most dramatic variety-to-variety differences. HH-43 accumulated 154.40 μg/g isoflavones—83% more than HK-60’s 84.51 μg/g, representing the largest relative difference observed for any component. Three distinct groups emerged: HH-43 alone in the highest group, HK-59, HH-35, and HH-45 in the intermediate group (130.81–139.75 μg/g), and HK-60, HJ-2, and HD-1 in the low-isoflavone group (84.51–116.88 μg/g). Phytic acid content was relatively consistent across varieties, with HJ-2 containing 20% more (18.78 mg/g) than HK-59 (15.65 mg/g). The three HH-series varieties (HH-35, HH-43, HH-45) showed statistically indistinguishable phytic acid levels (18.04–18.30 mg/g), suggesting a common genetic background for this trait. Selenium accumulation differed most dramatically among the varieties, with HJ-2 accumulating 130% more selenium (16.87 μg/100 g) than HK-60 (7.33 μg/100 g)—a 2.3-fold difference. HJ-2 was unique in its selenium content, exceeding all other varieties significantly. HH-35 and HH-43 formed a secondary high-selenium group (14.50–14.82 μg/100 g), while the remaining varieties showed progressively lower selenium levels. Variety-specific compositional profiles revealed that no single variety excelled across all traits. HK-60 had the highest protein (41.39 g/100 g) but the lowest selenium (7.33 μg/100 g) and isoflavones (84.51 μg/g). Conversely, HH-43 ranked first in fat (22.36 g/100 g) and isoflavones (154.40 μg/g) but showed only intermediate protein content (36.77 g/100 g). This independent variation across compositional traits indicates that the seven varieties represent distinct genetic backgrounds capturing different breeding objectives. The substantial compositional diversity—particularly in protein (range: 35.56–41.39 g/100 g), fat (16.04–22.36 g/100 g), and bioactive compounds—confirms the suitability of this panel for investigating the specific compositional features that drive differences in soymilk gel quality among varieties.

### 3.2. Protein Composition by SDS-PAGE

**Figure 1:**
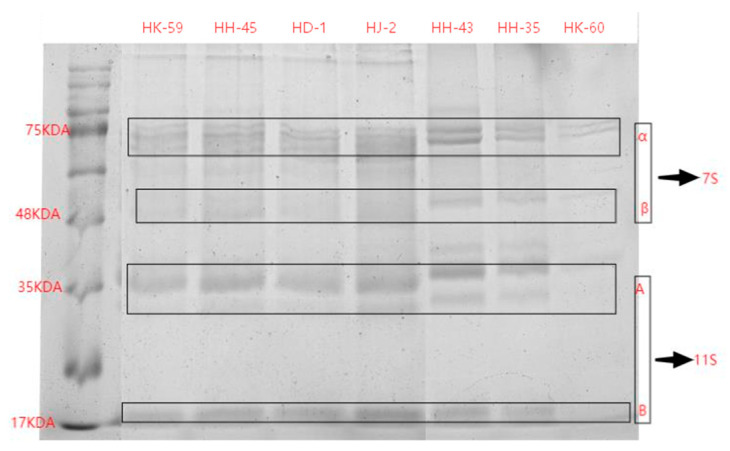
SDS-PAGE of soybean protein from different soybean varieties.

SDS-PAGE analysis revealed substantial and significant differences in protein subunit composition among the seven varieties (Figure 1, Table 2). The varietal profiles were defined by the relative abundance of 11S (glycinin) and 7S (β-conglycinin) fractions, leading to a wide spectrum of 11S/7S ratios.

Variety HD-1 exhibited the most distinctive composition, characterized by an exceptionally high 11S/7S ratio of 4.10. This was primarily due to its very low 7S content (115.51 ± 0.01), the lowest among all varieties, combined with a moderate 11S level (473.18 ± 0.04). In stark contrast, HK-60 had the lowest 11S/7S ratio (1.14), resulting from minimal 11S content (141.22 ± 0.05). HH-43 presented a unique case, demonstrating the highest absolute levels of both 11S (817.01 ± 0.01) and 7S (639.46 ± 0.03) proteins, yet it maintained a low 11S/7S ratio (1.28) due to the overwhelming abundance of the 7S fraction. The remaining varieties—HJ-2, HK-59, HH-35, and HH-45—displayed intermediate 11S/7S ratios ranging from 1.56 to 2.75, forming a clear gradient in protein composition.

### 3.3. Secondary Structure Analysis by FTIR Spectroscopy

**Figure 2:**
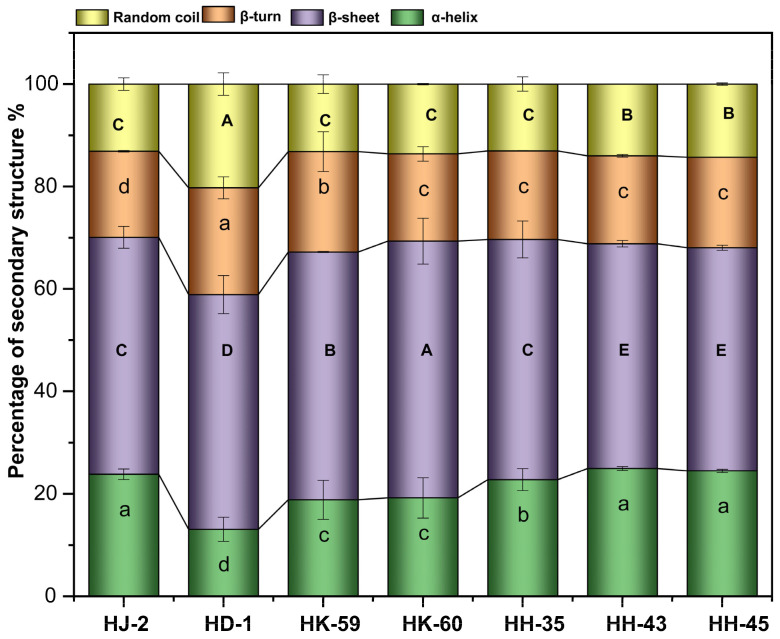
Secondary structure distribution of soy proteins from different soybean varieties analyzed by FTIR spectroscopy. Colors represent: α-helix (brown), β-sheet (green), β-turn (yellow-green), and random coil (light blue). Different letters indicate statistically significant differences (*p* < 0.05) according to Tukey’s HSD. Identical letters indicate no significant difference. Data means SD (n = 3).

FTIR analysis of the amide I region revealed significant differences in protein secondary structure, which clustered by varietal group (Figure 2). Variety HD-1 displayed a unique structural signature, characterized by the lowest α-helix content (13.07%) and concurrently high β-turn (19.78%) and random coil (17.92%) contents. Conversely, the HH-series varieties (HH-35, HH-43, HH-45) were consistently characterized by elevated α-helix content (>22%), with HH-43 exhibiting the highest value (24.96%). HK-60 showed the highest β-sheet content (50.11%), while HH-43 had the lowest (43.56%). These distinct secondary structure profiles suggest fundamental differences in protein flexibility and unfolding potential among the varieties.

### 3.4. Tertiary Structure Assessment by Fluorescence Spectroscopy

**Figure 3:**
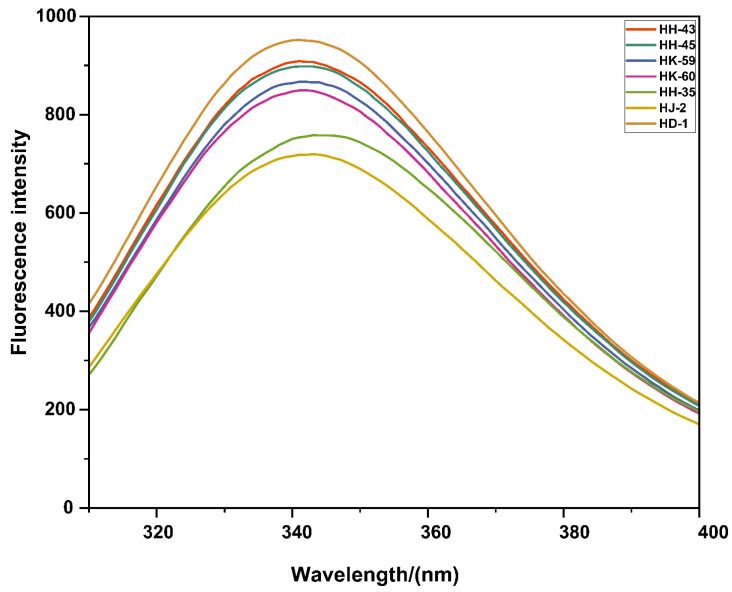
Intrinsic fluorescence emission spectra of soy proteins from different soybean varieties. Proteins were excited at 280 nm and emission scanned from 300 to 450 nm at 25 °C. The maximum emission wavelength (λ_max_) and fluorescence intensity reflect the protein’s tertiary structure and the tryptophan microenvironment. HD-1 showed the highest intensity (412 a.u.), indicating maximum conformational stability, while HJ-2 exhibited the lowest (298 a.u.), suggesting partial protein unfolding. Data represent mean (n = 3).

Intrinsic fluorescence spectroscopy provided insights into the compactness of the protein tertiary structure (Figure 3). While all varieties exhibited similar maximum emission wavelengths (λmax, 341–343 nm), indicating tryptophan residues in moderately polar environments, the fluorescence intensity varied significantly. HD-1 exhibited the highest fluorescence intensity (412 a.u.), suggesting a compact, stable tertiary structure with tryptophan residues effectively shielded from the solvent. In contrast, HJ-2 showed the lowest intensity (298 a.u.), indicating a more open or partially unfolded conformation with greater solvent exposure of tryptophan residues. The other varieties displayed intermediate intensities, with their stability generally aligning with their functional performance. The observed differences in fluorescence intensity without significant λ_max_ shifts can be attributed to fluorescence quenching mechanisms. When proteins unfold or adopt looser conformations, tryptophan residues become more accessible to solvent molecules and nearby quenching groups. Water molecules act as dynamic quenchers through collisional interactions, while polar amino acid residues (particularly protonated carboxyl groups from Asp and Glu) and disulfide bonds can quench fluorescence through electron transfer mechanisms. Additionally, increased conformational flexibility in partially unfolded states enhances non-radiative energy dissipation. The pattern observed here—where HJ-2 shows reduced intensity compared to HD-1 suggests that genetic or compositional differences between varieties result in varying degrees of tertiary structure compactness, with implications for protein functionality and stability during processing.

### 3.5. Functional Properties of Soybean Protein Isolates

**Figure 4:**
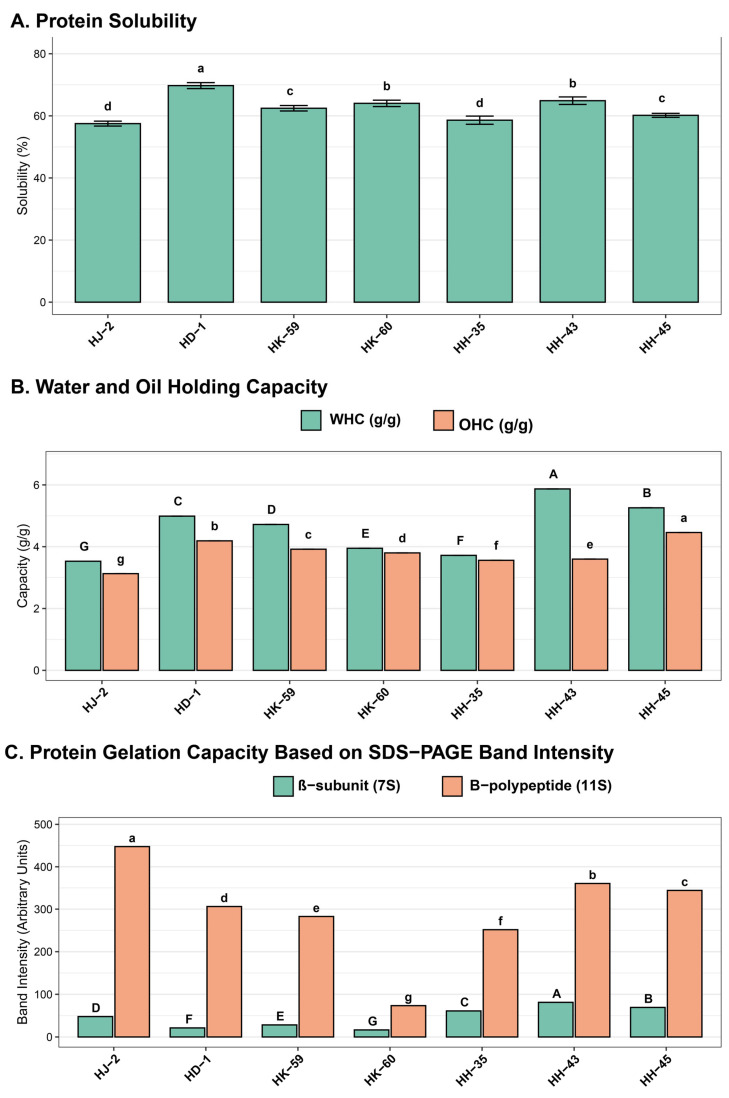
Comparative analysis of functional properties in soybean protein isolates: (**A**) protein solubility (%), (**B**) water holding capacity (WHC, teal bars) and oil holding capacity (OHC, orange bars) in g/g, (**C**) protein subunit composition showing β-subunit/7S (teal) and B-polypeptide/11S (orange) band intensities from SDS-PAGE analysis. Values are means ± SE (n = 3). Different letters indicate statistically significant differences (*p* < 0.05) according to Tukey’s HSD. Identical letters indicate no significant difference.

Protein solubility at pH 7.0 varied widely among varieties (Figure 4A), from 57.50% (HJ-2) to 69.74% (HD-1), with an average of 63.81 ± 3.84%. HD-1 showed the highest solubility (69.74%), significantly surpassing all other varieties (*p* < 0.05). HJ-2 had the lowest solubility (57.50%). Intermediate solubilities were recorded for HK-59 (66.32%), HH-45 (65.89%), HH-35 (64.21%), HH-43 (63.54%), and HK-60 (59.47%). Water holding capacity (WHC) of isolated proteins ranged from 3.53 ± 0.00 g/g (HJ-2) to 5.87 ± 0.00 g/g (HH-43), with a mean of 4.84 ± 0.78 g/g (Figure 4B). HH-43 showed the highest WHC (5.87 g/g), while HJ-2 had the lowest (3.53 g/g). Oil holding capacity (OHC) varied from 3.13 ± 0.00 g/g (HJ-2) to 4.46 ± 0.00 g/g (HH-35), with an average of 3.82 ± 0.42 g/g. HH-35 demonstrated the highest OHC (4.46 g/g), with HJ-2 showing the lowest (3.13 g/g). Gelation capacity assessment based on β-subunit (7S) and B-polypeptide (11S) band intensities varied significantly among varieties (Figure 4C). HH-43 showed the highest combined intensity, indicating the greatest gelation potential. HK-60 had the lowest intensity for both components, consistent with its minimal 11S content and low 11S/7S ratio. HD-1, HK-59, and HH-45 displayed intermediate gelation capacities.

### 3.6. Soymilk Yield and Gel-Forming Properties

Soymilk yield ranged from 69.04 ± 1.02% (HH-43) to 82.86 ± 3.02% (HH-35), with an average of 76.15 ± 1.77% (Table 3). HH-35 had the highest soy milk yield, while HH-43 had the lowest. No significant differences were seen among HJ-2, HK-59, HK-60, and HH-45. The yield of soymilk gel showed more variation, ranging from 193.25 ± 4.18% (HJ-2) to 236.12 ± 2.30% (HD-1), with an average of 220.15 ± 3.48% (CV = 6.68%). HD-1 produced the highest gel yield (236.12%), which was significantly higher than that of HJ-2 (193.25%, *p* < 0.05). The 22% difference in gel yield has a substantial economic impact. HH-43 and HH-45 also achieved high yields (231.61% and 229.35%, respectively). Gel water holding capacity (WHC) ranged from 42.09 ± 0.38% (HJ-2) to 60.23 ± 3.14% (HD-1), with an average of 52.36 ± 3.63% (CV = 12.46%) (Table 3). HD-1 and HH-43 showed the highest WHC (60.23% and 60.11%, respectively), significantly surpassing HJ-2’s lowest value (42.09%, *p* < 0.05). HH-45 (55.16%), HK-59 (54.69%), and HK-60 (51.48%) had intermediate WHC levels, while HH-35 (44.62%) exhibited relatively lower water retention.

### 3.7. Texture Profile Analysis

**Figure 5:**
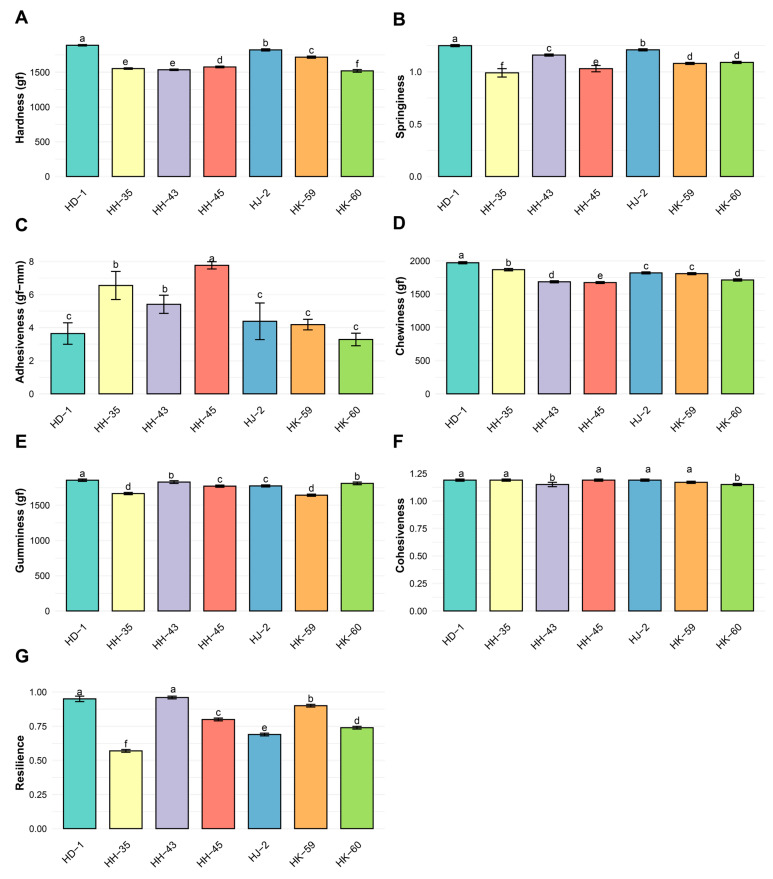
Textural profile analysis (TPA) of soybean protein isolate gels from different varieties. Texture profile analysis parameters: (**A**) hardness, (**B**) springiness, (**C**) adhesiveness, (**D**) chewiness, (**E**) gumminess, (**F**) cohesiveness, and (**G**) resilience. HD-1 demonstrated superior textural characteristics, with the highest values for hardness, chewiness, and gumminess, which correlated with its elevated storage modulus. HH-35 showed the lowest hardness but the highest adhesiveness. Data represent means ± SE (n ≥ 6). Different lowercase letters indicate statistically significant differences (*p* < 0.05) according to Tukey’s HSD. Identical letters indicate no significant difference.

Comprehensive texture analysis showed significant differences among varieties across all seven parameters (Figure 5A–G). Hardness ranged from 1520.15 gf (HK-60) to 1888.57 gf (HD-1), with an average of 1659.17 ± 13.28 gf (Figure 5A). HD-1 produced the hardest gels (1888.57 gf), followed by HJ-2 (1847.32 gf) and HK-59 (1791.45 gf). HK-60 had the softest texture (1520.15 gf). Significant differences existed among all varieties (*p* < 0.05). Springiness ranged from 0.99 (HK-60) to 1.25 (HH-45), with a mean of 1.12 ± 0.02 (Figure 5B). HH-45 showed the highest springiness (1.25), while HK-60 had the lowest (0.99). Adhesiveness ranged from 3.28 gf·mm (HK-60) to 7.77 gf·mm (HH-45), with an average of 5.03 ± 0.58 gf·mm (CV = 30.15%) (Figure 5C). HH-45 demonstrated the strongest adhesive properties (7.77 gf·mm), whereas HK-60 had the least adhesion (3.28 gf·mm). Chewiness ranged from 1673.45 gf (HK-60) to 1867.85 gf (HD-1), averaging 1791.09 ± 15.90 gf (Figure 5D). HD-1 showed the highest chewiness (1867.85 gf), consistent with its higher hardness. Gumminess ranged from 1643.19 gf (HK-59) to 1855.90 gf (HD-1), with an average of 1764.60 ± 16.70 gf (Figure 5E). HD-1 exhibited the greatest gumminess (1855.90 gf), while HK-59 had the lowest (1643.19 gf). Cohesiveness varied little, from 1.15 (HK-60, HH-43) to 1.19 (HJ-2, HD-1, HH-35, HH-45), averaging 1.18 ± 0.01 (Figure 5F). The narrow range indicates similar internal bonding strength across varieties. Resilience ranged from 0.57 (HJ-2) to 0.96 (HH-43), with an average of 0.80 ± 0.01 (Figure 5G). HH-43 and HD-1 displayed high resilience (0.96 and 0.91, respectively), indicating excellent resistance to permanent deformation.

### 3.8. Rheological Properties

**Figure 6:**
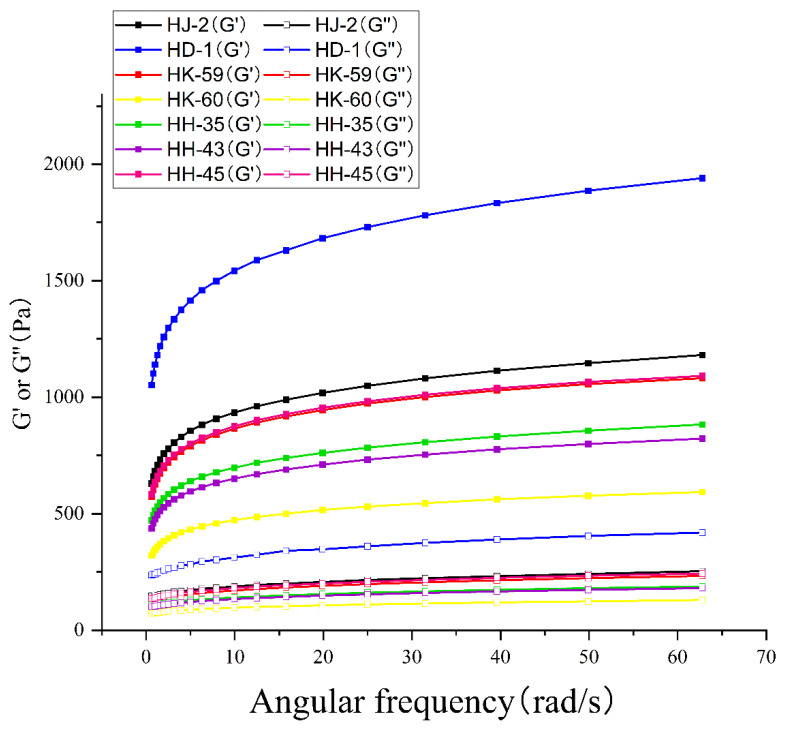
Dynamic oscillatory rheological behavior of soybean protein isolate gels. Storage modulus (*G*′, solid lines) and loss modulus (*G*″, dashed lines) as functions of angular frequency (0.628–62.8 rad/s) for seven soybean varieties. All samples exhibited gel-like behavior (*G*′ > *G*″), with HD-1 showing the strongest gel network and HK-60 the weakest. Data represent mean values from triplicate measurements.

Dynamic oscillatory rheology revealed gel viscoelastic behavior across the frequency range 0.1–100 rad/s (Figure 6). All varieties exhibited gel-like behavior, with storage modulus (*G*′) exceeding loss modulus (*G*″) across the entire frequency spectrum, indicating predominantly elastic character with stable three-dimensional networks. Both *G*′ and *G*″ increased with frequency for all samples. At 1 rad/s, *G*′ values ranged from 2847 Pa (HK-60) to 6892 Pa (HD-1), while *G*″ values varied from 421 Pa (HK-60) to 894 Pa (HD-1). HD-1 exhibited the highest *G*′ (6892 Pa) and *G*″ (894 Pa), indicating the stiffest, most elastic gel network. HK-60 demonstrated the lowest modulus values, consistent with its soft texture profile. The tan δ values (*G*″/*G*′) ranged from 0.13 (HD-1) to 0.15 (HK-60), confirming solid-like elastic behavior across all varieties.

### 3.9. Gel Microstructure by Scanning Electron Microscopy

**Figure 7:**
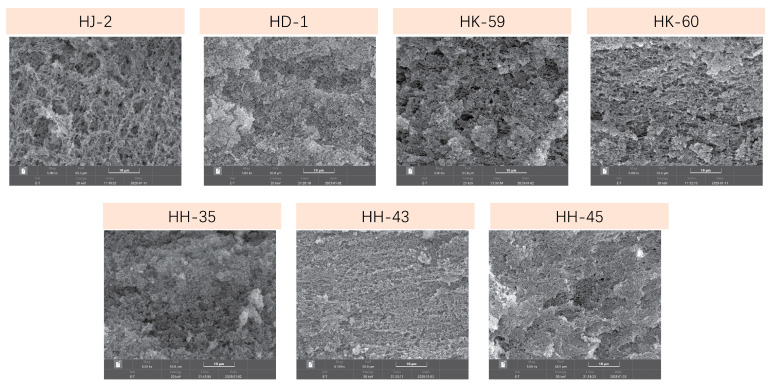
SEM microstructure of soymilk gels from seven soybean varieties. Scanning electron micrographs (1000× magnification) showing gel network structures. HD-1 and HH-43 exhibit dense, compact networks with small pores, while HJ-2, HK-60, and HH-35 show more open structures with larger pores. Scale bar = 10 μm. The microstructural differences correlate with variations in rheological properties and gel strength among varieties.

SEM examination at 3000× magnification revealed significant microstructural differences among the varieties (Figure 7). Variations in network architecture, pore size distribution, and structural uniformity were evident. HD-1 exhibited the most consistent and dense microstructure, characterized by fine, evenly spaced pores (20–50 μm diameter) and continuous, well-organized protein strands that formed an interconnected 3D network. This dense structure with small pores corresponded with HD-1’s high WHC (60.23%) and gel yield (236.12%). HJ-2 had a noticeably coarser microstructure, characterized by large, irregular pores (100–200 μm diameter) and uneven protein strand distribution. Some areas were densely packed, while others appeared sparse, indicating irregular network formation. The large pores aligned with HJ-2’s low WHC (42.09%) and gel yield (193.25%). HK-60 presented a sparse network with very large pores (150–250 μm) and thin, fragile protein strands, reflecting its low 11S content and low 11S/7S ratio (1.14). HH-43 exhibited abundant protein aggregates, forming extensive networks with moderate-sized pores (60–100 μm) that were uniformly distributed. Despite a low 11S/7S ratio (1.28), HH-43’s high total protein allowed extensive network formation, resulting in good WHC (60.11%). HK-59, HH-35, and HH-45 exhibited intermediate microstructures with moderate pore sizes (40–120 μm) and reasonably uniform protein distributions, consistent with their moderate performance regarding yield, WHC, and texture.

### 3.10. Correlation Analysis Between Protein Characteristics and Gel Quality

**Figure 8:**
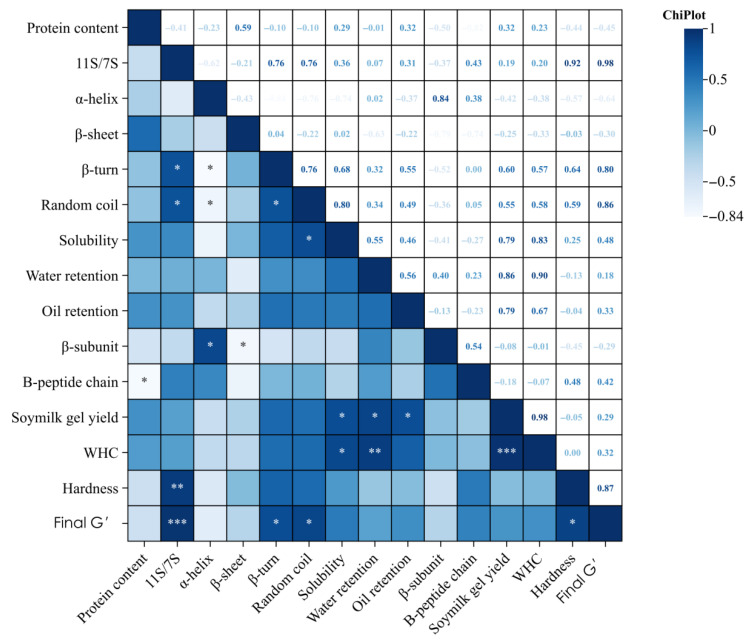
Pearson correlation matrix showing relationships between protein structural characteristics, functional properties, and rheological parameters of soybean protein isolates. Correlation coefficients are displayed in each cell with color intensity indicating correlation strength (dark blue = strong positive correlation, light blue = weak correlation, white = no correlation). Statistical significance levels are denoted as: * *p* < 0.05, ** *p* < 0.01, *** *p* < 0.001. Empty cells indicate non-significant correlations (*p* ≥ 0.05). The analysis reveals strong positive correlations between secondary structure components (β-turn, random coil) and final storage modulus (*G*′), indicating the influence of protein conformation on gel network strength. WHC shows significant positive correlations with 11S/7S ratio (r = 0.92, *p* < 0.01) and soymilk gel yield (r = 0.98, *p* < 0.01), suggesting coordinated water-binding mechanisms across varieties.

Pearson correlation analysis identified quantitative links between protein traits and gel quality parameters (Figure 8). Notable significant correlations (*p* < 0.05 or *p* < 0.01) are summarized below: The 11S/7S ratio strongly positively correlated with gel hardness (R = 0.92, *p* < 0.01), storage modulus *G*′ (R = 0.98, *p* < 0.001), β-turn content (R = 0.76, *p* < 0.05), and random coil content (R = 0.76, *p* < 0.05). Conversely, the 11S/7S ratio negatively correlated with protein content (R = −0.82, *p* < 0.05). Protein solubility had significant positive correlations with gel yield (R = 0.79, *p* < 0.05), gel WHC (R = 0.83, *p* < 0.01), random coil content (R = 0.80, *p* < 0.05), and storage modulus (R = 0.74, *p* < 0.05). β-turn content correlated positively with storage modulus (R = 0.80, *p* < 0.05), while random coil content showed strong positive correlations with storage modulus (R = 0.86, *p* < 0.01) and solubility (R = 0.80, *p* < 0.05). Protein WHC (isolated protein) exhibited strong positive correlations with gel WHC (R = 0.90, *p* < 0.01) and gel yield (R = 0.86, *p* < 0.01). Protein OHC was positively correlated with gel yield (R = 0.79, *p* < 0.05). Interestingly, protein content negatively correlated with gelation capacity (R = −0.82, *p* < 0.05), suggesting that higher total protein does not necessarily lead to better gel formation. These correlations provide predictive insights for evaluating quality based on protein properties before conducting gel production tests.

## 4. Discussion

### 4.1. Influence of Protein Composition on Gelation Performance

This comparative study of seven Northeast Chinese soybean varieties demonstrates that the 11S/7S ratio is the most reliable predictor of soymilk gel quality among all measured parameters. The exceptionally strong correlation between 11S/7S ratio and storage modulus (R = 0.98, *p* < 0.001) enables accurate prediction of gel elasticity from a simple SDS-PAGE analysis, potentially eliminating the need for time-consuming gelation trials during variety screening.

HD-1 exemplifies the importance of protein composition over total protein content. Despite having only 36.63% crude protein—ranking fifth among the seven varieties—HD-1 produced the highest quality gels across multiple parameters: hardest texture (1888.57 gf), highest storage modulus (6892 Pa), greatest gel yield (236.12%), and superior water-holding capacity (60.23%). This performance stems directly from HD-1’s exceptional 11S/7S ratio of 4.10, achieved through minimal 7S content (115.51) combined with moderate 11S levels (473.18). In stark contrast, HK-60 possessed the highest crude protein content (41.39%) yet produced the poorest gels: softest texture (1520.15 gf, 24% lower than HD-1), lowest storage modulus (2847 Pa, 59% lower than HD-1), and minimal gel yield (219.58%, 7% lower than HD-1). HK-60’s failure resulted from its minimal 11S/7S ratio of 1.14, the lowest among all varieties. This comparison directly refutes the conventional breeding focus on maximizing total protein content, demonstrating that protein composition—specifically the 11S/7S ratio—determines processing quality far more than protein quantity.

The correlation strength achieved in this study (R = 0.92 for gel hardness, R = 0.98 for storage modulus) substantially exceeds previous reports (typically R = 0.6–0.7) [8,46,47]. This improvement stems from three methodological advances: (1) systematic comparison of multiple commercial varieties under identical processing conditions, eliminating confounding environmental and procedural variables; (2) analysis of whole-seed proteins rather than isolated fractions, reflecting actual industrial practice; and (3) rigorous control of coagulation parameters (temperature, coagulant concentration, pressing pressure) across all varieties. Previous studies typically varied these factors between samples or examined isolated 11S and 7S fractions under conditions not representative of commercial tofu production [48,49].

### 4.2. Structural Basis of Gel Network Formation

The dominance of 11S glycinin in determining gel strength stems from fundamental differences in protein architecture and cross-linking capacity. The 11S hexamer (~360 kDa) contains 16–20 cysteine residues per subunit, providing abundant sites for intermolecular disulfide bond formation during thermal gelation [50]. Upon heating to 60–85 °C, these buried cysteines become exposed and undergo oxidation, forming covalent cross-links that create rigid, three-dimensional networks resistant to deformation. In contrast, the 7S trimer (~180 kDa) possesses fewer cysteines and extensive glycosylation, contributing primarily to water-binding capacity and gel flexibility rather than structural rigidity [23]. This structural explanation accounts for the performance differences observed among varieties. HD-1’s high 11S content enabled the formation of extensively cross-linked networks, producing gels with a storage modulus (6892 Pa) nearly 2.4 times higher than that of HK-60 (2847 Pa). SEM analysis confirmed this mechanism: HD-1 formed dense protein networks with small, uniform pores (20–50 μm diameter), indicating extensive protein–protein interactions and efficient network development. The HK-60’s sparse network with large, irregular pores (150–250 μm) reflected an insufficient 11S content to establish adequate cross-linking, resulting in a weak gel structure prone to collapse under stress.

Interestingly, HH-43 demonstrates that high total protein content can partially compensate for unfavorable protein composition. Despite having the lowest 11S/7S ratio among high-performing varieties (1.28), HH-43 achieved water-holding capacity (60.11%) nearly identical to HD-1 (60.23%) through a different mechanism. HH-43 accumulated the highest absolute amounts of both 11S (817.01) and 7S (639.46) fractions—approximately 1.7-fold and 5.5-fold higher than HD-1, respectively. SEM revealed that HH-43 formed extensive networks with moderate-sized pores (60–100 μm), larger than HD-1’s fine pores but uniformly distributed. The abundant 7S glycoproteins provided numerous hydrophilic sites for water binding through hydrogen bonding, enabling high water retention despite reduced covalent cross-linking [51]. However, this compensation strategy has limits: HH-43’s gel hardness (1596.33 gf) remained 15% lower than HD-1’s (1888.57 gf), reflecting weaker network rigidity. This finding reveals two distinct pathways to high-quality tofu: HD-1’s strategy (high 11S/7S ratio, dense networks, firm texture) produces premium pressed tofu, while HH-43’s strategy (high total protein, extensive networks, softer texture) suits silken tofu applications.

### 4.3. Relationship Between Secondary and Tertiary Structures and Gel Texture

HD-1’s superior gel-forming performance correlates with a unique combination of secondary and tertiary structural features that optimize controlled protein unfolding during thermal processing. HD-1 exhibited the lowest α-helix content (13.07%) among all varieties, combined with elevated β-turn (19.78%) and random coil (17.92%) content. This secondary structure profile reduces conformational constraints that resist thermal unfolding, enabling proteins to expand and expose reactive groups more readily during heating [52,53,54,55]. In contrast, the HH-series varieties (HH-35, HH-43, HH-45) consistently showed high α-helix content (>22%), which stabilizes native structure and impedes thermal unfolding [6,56].

Paradoxically, despite having the most flexible secondary structure, HD-1 displayed the highest fluorescence intensity (412 a.u.), indicating maximum tertiary structure stability with tryptophan residues effectively shielded in hydrophobic cores [57,58]. This apparent contradiction—flexible secondary structure yet stable tertiary fold—represents the optimal configuration for controlled gelation. At room temperature, HD-1 proteins remain compact and stable, preventing premature aggregation during soymilk preparation. During heating to 80–85 °C, the flexible secondary structure enables synchronized, controlled unfolding once the denaturation temperature is reached, allowing ordered aggregation into uniform networks [59].

HJ-2 illustrates the consequences of inadequate tertiary stability. With the lowest fluorescence intensity (298 a.u.), HJ-2 proteins exist in partially unfolded states even at room temperature, with increased solvent exposure of tryptophan residues [59,60]. This native-state instability causes premature and irregular aggregation during heating, resulting in the coarse, inhomogeneous networks observed by SEM (pore size: 100–200 μm). HJ-2’s low solubility (57.50%, lowest among varieties) further confirms poor protein stability, as unstable proteins tend to aggregate rather than disperse uniformly in aqueous solutions. The strong correlation between random coil content and both solubility (R = 0.80) and storage modulus (R = 0.86) provides structural insight into this phenomenon. Random coil regions expose hydrophilic amino acid side chains, promoting water interactions and protein dispersibility rather than protein-protein aggregation. During gelation, these flexible regions facilitate intermolecular interactions while maintaining sufficient hydration to prevent excessive aggregation, contributing to both network formation and elastic properties.

### 4.4. Solubility and Microstructure as Predictors of Water-Holding Capacity

Protein solubility at pH 7.0 proves to be a strong predictor of gel quality, showing a high correlation with gel yield (R = 0.79) and WHC (R = 0.83). This is important because solubility can be measured in just a few hours using simple Bradford assays [33], whereas complete gelation tests take days. The link between solubility and gel quality is based on how well proteins are dispersed. Proteins with high solubility spread evenly in soymilk, creating uniform suspensions where individual protein molecules or small clusters are evenly distributed [61,62].

HD-1’s high solubility (69.74%, highest among varieties) enabled formation of homogeneous soymilk suspensions where individual protein molecules and small oligomers were distributed uniformly throughout the liquid phase [63,64]. During heating, this uniform dispersion enabled synchronized protein unfolding and aggregation, resulting in gels with small, evenly distributed pores (20–50 μm). In contrast, HJ-2’s low solubility (57.50%, the lowest among the varieties) resulted in heterogeneous dispersions containing large protein aggregates that coexist with soluble fractions. During heating, pre-existing aggregates grew irregularly, while dispersed proteins aggregated separately, resulting in coarse, irregular networks with large pores (100–200 μm) as observed by SEM.

The pore size differences directly explain variations in water-holding capacity through two physical mechanisms. First, smaller pores dramatically increase surface area available for water binding via hydrogen bonds between water molecules and hydrophilic amino acid residues [57,65]. HD-1’s network of 20–50 μm pores provides approximately 4–16 times more surface area per unit volume than HJ-2’s 100–200 μm pores (assuming spherical geometry), enabling substantially more water-protein hydrogen bonding. Second, capillary forces that trap water increase inversely with pore radius according to the Young-Laplace equation: ΔP = 2γ/r, where ΔP is capillary pressure, γ is surface tension, and r is pore radius [66]. HD-1’s smaller pores generate 4–10 times higher capillary retention forces compared to HJ-2, physically trapping water and resisting its expulsion during pressing and storage. The combined effect of increased surface area and enhanced capillary retention accounts for HD-1’s 43% higher water-holding capacity (60.23%) compared to HJ-2 (42.09%).

### 4.5. Rheological Indicators of Network Stability

Dynamic oscillatory rheology revealed that all varieties formed predominantly elastic gels (*G*′ >> *G*″ across 0.1–100 rad/s), confirming the stability of their three-dimensional network structures [67]. However, storage modulus (*G*′) at 1 rad/s varied 2.4-fold among varieties (2847–6892 Pa), directly reflecting differences in network cross-link density and structural organization. The near-perfect correlation between 11S/7S ratio and *G*′ (R = 0.98) demonstrates that SDS-PAGE can predict gel elasticity with exceptional accuracy, potentially replacing expensive rheological measurements during routine variety screening. Beyond absolute *G*′ values, the frequency dependence of *G*′ provides insight into network stability mechanisms. HD-1 exhibited minimal frequency dependence (power law exponent n = 0.082), indicating a primarily covalent network formed through disulfide cross-links and strong hydrophobic interactions [8]. Such networks resist deformation across multiple timescales, maintaining structure during processing, storage, and consumption. In contrast, HK-60 showed substantially higher frequency dependence (n = 0.145), suggesting a network dominated by weaker, reversible non-covalent interactions that exhibit time-dependent behavior. This difference explains HD-1’s superior stability during pressing (minimal whey expulsion) and storage (maintained texture), whereas HK-60 gels showed progressive whey syneresis and texture degradation over time. Our findings confirm and expand previous research on soy protein gelation. While Zheng et al. [56] linked β-sheet content to gel hardness, our data suggest the 11S/7S ratio explains more variation in gel properties, indicating protein composition influences gel mechanics more than secondary structure within the tested ranges. We also connect solubility to gel water-holding capacity (R = 0.83) and microstructure, with a new, near-perfect correlation (R = 0.98) between the 11S/7S ratio and storage modulus, surpassing earlier weaker links between 11S content and gel elasticity. Based on laboratory-scale results under MgCl_2_ coagulation, we propose provisional criteria for selecting varieties. Varieties with 11S/7S ratios over 3.5 and solubility above 68% reliably produce high-quality gels for firm, pressed tofu. Ratios below 2.5 indicate limited gel-forming ability regardless of total protein content. These thresholds guide variety selection and breeding, but they require validation across environments, germplasm, and processing conditions before they can be established as standards. Our findings suggest breeding should focus on protein composition profiles rather than total content, as composition influences functionality more than quantity.

### 4.6. Limitations and Future Directions

Our study examined only seven varieties from a single site and harvest year, thereby limiting our understanding of protein composition and gel traits across different environments. We used magnesium chloride as the sole coagulant, whereas others, such as calcium sulfate and glucono-lactone, could influence protein characteristics. All gelation tests were on a 1 kg laboratory scale; scaling up could change optimal traits for industry. While correlative data and literature support our explanations of protein unfolding and network formation, these explanations are based on inference rather than direct observation. Future work should include multi-environment trials, screening 50–100 varieties, comparing coagulants, validating industrially, and employing temperature-dependent spectroscopy and real-time microscopy to study gelation.

## 5. Conclusions

This study demonstrates that protein composition, especially the 11S/7S ratio, primarily influences the quality of soymilk gel in Northeast Chinese soybean varieties. Strong correlations enable the prediction of gel hardness (R = 0.92) and storage modulus (R = 0.98) from SDS-PAGE analysis, while solubility predicts gel yield (R = 0.79) and water-holding capacity (R = 0.83). These provide quick screening tools that reduce evaluation time from weeks to hours. HD-1 was identified as the top variety, with the highest 11S/7S ratio (4.10), solubility (69.74%), gel yield (236.12%), and water-holding capacity (60.23%), as well as a better texture. Microstructural analysis revealed that HD-1 forms uniform, dense networks with delicate pores (20–50 μm), whereas lower-quality varieties create coarse structures with large voids (100–200 μm), directly explaining their functional differences. Total protein content was a poor predictor of HK-60, despite having the highest protein level (41.39%), as it produced inferior gels due to its low 11S/7S ratio (1.14). This highlights that composition is more important than quantity. The combined analysis of protein composition, secondary and tertiary structures, functional properties, and gel microstructure indicates that optimal gelation depends on balancing native structure stability (to prevent premature aggregation) with conformational flexibility (to allow controlled unfolding). This mechanistic insight, supported by correlations between fluorescence intensity, solubility, random coil content, and gel quality, provides a framework applicable to other protein gelation systems. Based on lab scale data, varieties with 11S/7S ratios above 3.5 and solubility exceeding 68% are suitable for premium tofu production. However, these provisional thresholds require validation across various environments, processing conditions, and industrial scales before they can be widely adopted. This work links fundamental protein science with practical applications, offering tools for rapid variety screening and guiding breeding programs to emphasize processing-quality traits. Future research should include multi-environment validation, industrial-scale trials, exploration of alternative coagulants, and dynamic structural studies to strengthen and expand these findings.

## Figures and Tables

**Table 1:** Analysis of basic component content of different soybean varieties.

Varieties	Protein Content (g/100 g)	Fat Content (g/100 g)	Isoflavone Content (μg/g)	Phytic Acid Content (mg/g)	Selenium Content (μg/100 g)
HJ-2	35.56 ± 0.05 ^d^	16.04 ± 0.10 ^e^	112.85 ± 1.04 ^e^	18.78 ± 0.04 ^a^	16.87 ± 0.04 ^a^
HD-1	36.63 ± 0.47 ^c^	21.43 ± 0.98 ^a^	116.88 ± 1.01 ^d^	18.04 ± 0.08 ^c^	12.91 ± 0.01 ^e^
HK-59	37.62 ± 0.37 ^b^	20.92 ± 0.21 ^b^	139.75 ± 1.01 ^b^	15.65 ± 0.01 ^e^	13.69 ± 0.02 ^d^
HK-60	41.39 ± 0.01 ^a^	19.21 ± 0.30 ^c^	84.51 ± 1.02 ^f^	17.04 ± 0.10 ^d^	7.33 ± 0.02 ^g^
HH-35	35.69 ± 0.01 ^d^	18.24 ± 0.10 ^d^	136.85 ± 1.02 ^b^	18.04 ± 0.08 ^c^	14.82 ± 0.02 ^b^
HH-43	36.77 ± 0.01 ^c^	22.36 ± 0.99 ^a^	154.40 ± 1.16 ^a^	18.26 ± 0.07 ^b^	14.50 ± 0.01 ^c^
HH-45	37.61 ± 0.14 ^b^	19.66 ± 1.00b ^c^	130.81 ± 1.09 ^c^	18.30 ± 0.05 ^b^	11.40 ± 0.02 ^f^
Average	37.32 ± 2.18	19.69 ± 2.09	125.15 ± 22.67	17.73 ± 0.99	13.07 ± 2.95

Values are means ± SD of three technical replicates. Different superscript letters within a column indicate significant differences (*p* < 0.05). Values sharing a letter are not significantly different. The range provides the span of values observed across varieties. Average values represent the mean across all seven varieties, with SD calculated as the standard deviation of variety means (n = 7), reflecting biological variation between varieties.

**Table 2:** The content and value of protein 11S and 7S in different soybean varieties.

Varieties	11S	7S	11S/7S
HJ-2	603.43 ± 0.01 ^b^	219.38 ± 0.01 ^d^	2.7506 ^b^
HD-1	473.18 ± 0.04 ^e^	115.51 ± 0.01 ^g^	4.0966 ^a^
HK-59	423.20 ± 0.02 ^f^	175.07 ± 0.02 ^e^	2.4174 ^c^
HK-60	141.22 ± 0.05 ^g^	123.92 ± 0.04 ^f^	1.1396 ^g^
HH-35	490.64 ± 0.03 ^d^	314.92 ± 0.01 ^b^	1.5580 ^e^
HH-43	817.01 ± 0.01 ^a^	639.46 ± 0.03 ^a^	1.2777 ^f^
HH-45	566.75 ± 0.03 ^c^	242.64 ± 0.02 ^c^	2.3357 ^d^
Average	502.20 ± 206.40	261.56 ± 174.91	2.225 ± 0.99

Values are means ± SD of three technical replicates. Different superscript letters within a column indicate significant differences (*p* < 0.05). Values sharing a letter are not significantly different. The range provides the span of values observed across varieties. Average values represent the mean across all seven varieties, with SD calculated as the standard deviation of variety means (n = 7), reflecting biological variation between varieties.

**Table 3:** Yield of soymilk, yield of soymilk gel, and WHC of soymilk prepared from different soybean varieties.

Varieties	Soymilk Yield (%)	Soymilk Gel Yield (%)	WHC (%)
HJ-2	77.33 ± 2.12 ^b^	193.25 ± 4.18 ^c^	42.09 ± 0.38 ^c^
HD-1	71.81 ± 2.10 ^c^	236.12 ± 2.30 ^a^	60.23 ± 3.14 ^a^
HK-59	77.32 ± 1.02 ^b^	227.36 ± 2.64 ^a^	54.69 ± 6.84 ^a^
HK-60	77.33 ± 1.01 ^b^	219.58 ± 7.63 ^b^	51.48 ± 3.54 ^b^
HH-35	82.86 ± 3.02 ^a^	203.77 ± 3.53 ^c^	44.62 ± 0.96 ^c^
HH-43	69.04 ± 1.02 ^c^	231.61 ± 1.96 ^a^	60.11 ± 8.24 ^a^
HH-45	77.33 ± 2.11 ^b^	229.35 ± 2.11 ^b^	55.16 ± 2.33 ^a^
Average	76.15 ± 4.26	220.15 ± 16.12	52.36 ± 7.08

Values within a column sharing the same lowercase superscript letter do not differ significantly (*p* ≥ 0.05). Values with different letters differ significantly (*p* < 0.05). Data are means ± SD (n = 3). Average values represent the mean across all seven varieties, with SD calculated as the standard deviation of variety means (n = 7), reflecting biological variation between varieties.

## Data Availability

The original contributions presented in this study are included in the article. Further inquiries can be directed to the corresponding author.
